# Epidemiological and genetic considerations in retinoblastoma

**Published:** 2018-06-03

**Authors:** Ido Didi Fabian, Elisabeth Rosser, Mandeep S Sagoo

**Affiliations:** 1 Consultant Ocular Oncologist: Ocular Oncology Service, Goldschleger Eye Institute, Sheba Medical Center, Tel Aviv, Israel.; 2 Consultant Clinical Geneticist: Clinical Genetics Unit, Great Ormond Street Hospital, Great Ormond Street, London, UK.; 3 Retinoblastoma Service: Royal London Hospital; Ocular Oncology Service NIHR Biomedical Research Centre for Ophthalmology, Moorfields Eye Hospital and UCL Institute of Ophthalmology, London, UK.

**Retinoblastoma is usually initiated by a random mutation of a gene in a retinal cell. It is important to try and recognise if the child has germline retinoblastoma, as this may affect both eyes of the child. Siblings and future children of the child with retinoblastoma are at greater risk of developing this cancer.**

## Incidence

Retinoblastoma (Rb) is the most common intraocular malignancy of childhood, but a relatively rare disease, occurring in approximately 1: 16,000–18,000 live births.^1^ Its incidence is uniform across populations, with no gender or ethnic predilection and no environmental or socio-economic factors. Worldwide, approximately 8,000 children develop Rb each year. Of these, over 80% are from low- to middle-income countries from Asia and Africa. Rb develops in early childhood, with the vast majority of cases presenting before the age of 5 years.

## Aetiology

**Figure 1:**
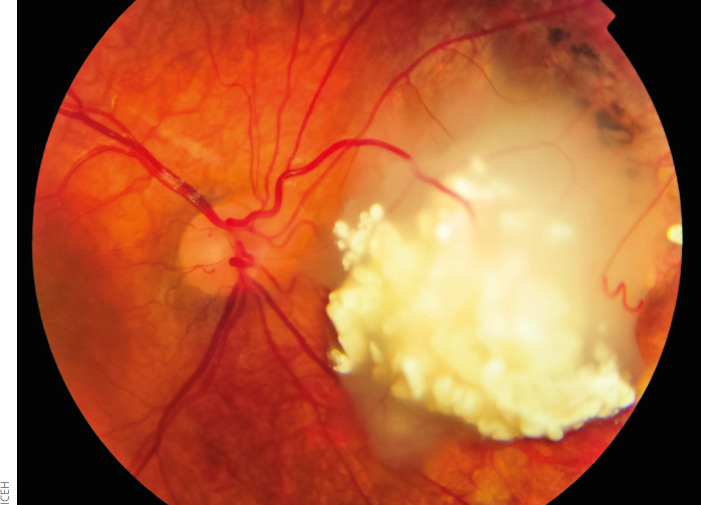
A right eye nasal retinocytoma incidentally found by an optometrist in an asymptomatic 8-year old boy with 20/20 vision. The patient was thereafter monitored in a specialised retinoblastoma service, with unchanged tumour on consecutive examinations.

Rb can be inherited or develop *de novo* (sporadic) in a child with no family history of Rb. The cancer can involve one or both eyes and may present in an asymmetrical manner, with different grade eyes at presentation or even a unilateral presentation, with disease developing in the other eye later. The disorder originates in a photoreceptor cell of the retina early in childhood. In most instances, there is a mutation in the *RB1* gene. *RB1* loss initially produces a retinoma (Figure 1), the benign precursor of Rb, and causes genomic instability that subsequently leads to the cancerous tumour known as retinoblastoma. Interestingly, retinomas should be looked for and can be found in the parents of affected children, confirming that the disease is inherited in that family.

**Figure 2:**
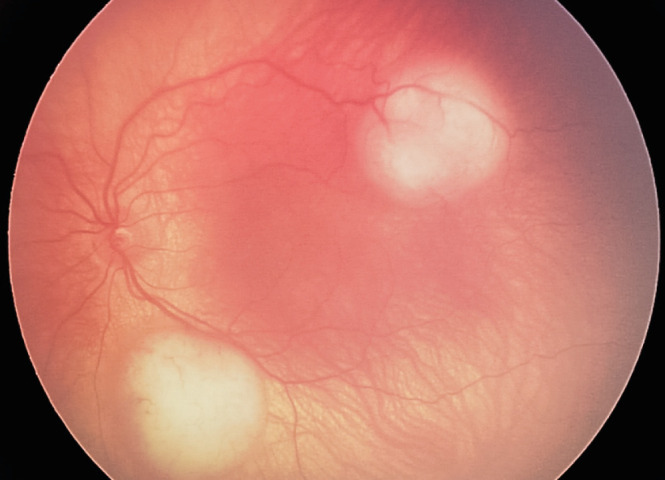
Multifocal retinoblastoma in the left eye of a 1-year old child.

## Characteristics of hereditary and sporadic disease

Patients diagnosed with Rb are categorised by whether the mutation is germline or non-germline (i.e. somatic). In germline disease, a single *RB1* allele is mutated in every cell of a child's body. An additional ‘hit’ in the second allele in the developing retina will result in clinical Rb. These children usually present with bilateral and multifocal disease (Figure 2) at a young age, median of 15 months, but can present with unliteral disease, albeit less frequently. A patient that presents with bilateral disease is 100% germline. However, it is estimated that 10–20% of unilateral cases are also germline, emphasising the importance of genetic testing in addition to clinical examination.

Somatic (non-germline) cases usually present at a later age (median: 24 months) with unilateral and unifocal disease. In order for the disease to develop in somatic cases, two consecutive ‘hits’ need to occur in a retinal cell, resulting in mutation of both *RB1* alleles and development of clinical Rb.

## Mosaicism

All heritable cases are germline, but not all germline cases have a familial history. This is because a mutation can occur at or after conception in an individual with no family history of Rb. Depending on the stage of development at which the mutation occurs, some of the foetus' cells will have a mutated *RB1* allele, and others will not, resulting in a mosaicism. Children with mosaicism are at increased risk of developing Rb. The disease in this scenario has no family history. The siblings of the affected child are not at risk, but offspring may be at risk and should therefore be screened soon after birth.

## Developing Rb genetics and counselling

It was long believed that mutated *RB1* genes are a prerequisite to develop Rb. Recently, however, researchers have found that Rb may arise even in the presence of non-mutated *RB1* genes when the MYCN oncogene is amplified.^2^ These cases are relatively rare, occurring in <3% of unilateral Rb cases, and present earlier, at a median age of 4.5 months.

**“Genetic testing is not available in all centres across the world, being particularly sparse in low-resource countries.”**

The field of Rb molecular genetics has evolved significantly since the *RB1* gene was cloned in the mid-1980's.^2^ Today, genetic laboratories are able to detect specific mutations and correlate them to the probability of developing Rb in an individual and her or his relatives. It has also set the basis for the development of screening programmes, which are discussed by Rosser et al in the current issue.

Knowledge of the genetic status has direct impact on the recommended screening frequency and also on the recommended screening protocol for siblings and offspring. Individuals harboring a germline mutation are also at risk of developing secondary non-Rb malignancies later in life, a risk that is further intensified if treated with external-beam radiotherapy, a treatment modality that used to be commonly used for Rb.^3^

Genetic testing, however, is not available in all centres across the world, being particularly sparse in low-resource countries. Much effort is put into improving Rb management and public health related to Rb in these countries. Genetic testing and screening will depend on genetic services being developed in these settings. Until then, clinicians should use epidemiological and clinical signs, including the age of presentation, laterality, tumour focality, presence of retinoma in a parent and family history of Rb, to counsel patients and their families.
